# Whole Transcriptome Analysis of *Aedes albopictus* Mosquito Head and Thorax Post-Chikungunya Virus Infection

**DOI:** 10.3390/pathogens8030132

**Published:** 2019-08-27

**Authors:** Ravi kiran Vedururu, Matthew J. Neave, Vinod Sundaramoorthy, Diane Green, Jennifer A. Harper, Paul R. Gorry, Jean-Bernard Duchemin, Prasad N. Paradkar

**Affiliations:** 1 CSIRO Health & Biosecurity, Australian Animal Health Laboratory, Geelong 3220, Australia; 2 School of Sciences, RMIT University, Bundoora 3083, Australia; 3 CSIRO, Australian Animal Health Laboratory, Geelong 3220, Australia; 4 School of Health and Biomedical Science, RMIT University, Bundoora 3083, Australia

**Keywords:** Chikungunya, *Aedes albopictus*, RNASeq, host–pathogen interactions, Bruton’s tyrosine kinase, *BTKi*

## Abstract

Chikungunya virus (CHIKV) is transmitted by *Aedes* mosquitoes and causes prolonged arthralgia in patients. After crossing the mosquito midgut barrier, the virus disseminates to tissues including the head and salivary glands. To better understand the interaction between *Aedes albopictus* and CHIKV, we performed RNASeq analysis on pools of mosquito heads and parts of the thorax 8 days post infection, which identified 159 differentially expressed transcripts in infected mosquitos compared to uninfected controls. After validation using RT-qPCR (reverse transcriptase-quantitative polymerase chain reaction), inhibitor of Bruton’s tyrosine kinase (*BTKi*), which has previously been shown to be anti-inflammatory in mammals after viral infection, was further evaluated for its functional significance. Knockdown of *BTKi* using double-stranded RNA in a mosquito cell line showed no significant difference in viral RNA or infectivity titer. However, *BTKi* gene knocked-down cells showed increased apoptosis 24 hours post-infection compared with control cells, suggesting involvement of *BTKi* in the mosquito response to viral infection. Since *BTK* in mammals promotes an inflammatory response and has been shown to be involved in osteoclastogenesis, a hallmark of CHIKV pathogenesis, our results suggest a possible conserved mechanism at play between mosquitoes and mammals. Taken together, these results will add to our understanding of *Aedes Albopictus* interactions with CHIKV.

## 1. Introduction

Chikungunya virus (CHIKV) is an enveloped, positive-sense RNA virus belonging to the Alphavirus genus in the Togaviridae family [1,2]. The virus causes a disease characterised by a high febrile illness and debilitating and protracted arthralgia [3,4,5].

Since the 2004–2005 outbreak in Reunion island, east of Africa, *Aedes albopictus* has played a significant role in expansion of CHIKV outbreaks around the world. A single mutation in the E1 viral gene has been shown to confer increased vector competence to this mosquito [6]. Phylogenetic analysis of CHIKV genome sequences has identified three distinct lineages, namely West African, East/Central/south African (ECSA) and Asian. The Indian ocean sub-lineage (IOL), which caused the 2004 Reunion Island outbreak, is considered to have evolved from within the ECSA clade. An American sub-lineage was identified to have emerged from within the Asian clade from an outbreak in the Caribbean and American states from around 2013. Virulence and pathogenicity of CHIKV varies among different lineages and clades with the West African lineage virus, which is considered to be more virulent than the Asian and American groups [7,8]. While most Alphaviruses are genetically similar, differences in 3’ untranslated regions and in proteins including E2 and nSP3 are thought to contribute significantly to their individual virulence and pathogenesis characteristics [8,9,10,11]. Antisera raised against one lineage of CHIKV has also been found to be protective against other lineages of CHIKV, suggesting a high level of serological conservation [8,12].

In the mosquito after blood-feeding, the virus needs to cross two critical barrier tissues, the midgut and salivary glands. The virus must infect and cross the epithelial cells before digestion of the blood meal and then overcome the midgut escape barriers to disseminate into the haemocoel, from where it reaches and infects other mosquito tissues, including the salivary glands [13]. The mosquito is considered to be infective once the virus is detected in the saliva [14,15]. 

Previous research has identified a number of genes involved in the early stages of *Aedes albopictus* infection with CHIKV [16]. However, to understand the interactions between the virus and mosquito at the transcriptomic level after dissemination of the virus, we performed RNASeq analysis of the head and anterior one-third part of the thorax (containing the salivary glands), collected 8 days post CHIKV infection (dpi). The results identified multiple differentially expressed genes involved in various biological and molecular cellular functions. Our results identified *BTKi* to be functionally significant in the mosquito response to CHIKV infection. 

## 2. Results

### 2.1. RNASeq and Differential Gene Expression (DGE) Analysis

To determine differentially expressed genes, two pools of head/thorax (six per pool) collected at 8 dpi from infected mosquitoes were created. For controls, one pool of head/thorax from uninfected mosquitoes was used. The three libraries yielded between 40.6 million and 47.15 million reads. After quality trimming and removing the reads mapping to the chicken genome (to remove read contributions by undigested chicken blood contamination), the remaining reads were aligned to the *Aedes albopictus* reference genome (Foshan strain genome sequence (AaloF1) from Vectorbase). The reads that did not map to the genome were then used to build a custom de novo transcriptome. The results confirmed the infection status of the two pools, and the control library contained no CHIKV reads as expected (reference sequence: MH229986) (Table 1).

**Figure 1:**
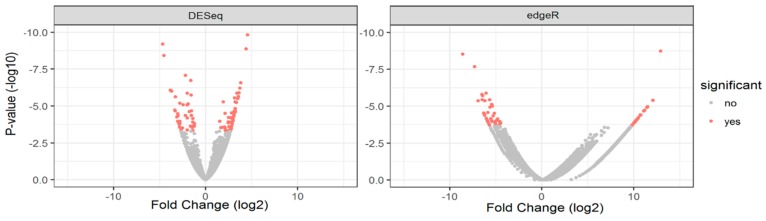
Volcano plots from DESeq2 and edgeR.

Several differentially expressed genes were identified using both DESeq2 (for reads aligned to the RefSeq genome) and edgeR (for the de novo transcriptome built with unaligned reads) (Table 2), and the data was visualised as Volcano plots (Figure 1). The full list of genes and transcripts differentially expressed with *p*-values (and FDR (False Discovery Rate)) of less than 0.05 is provided in Supplementary File S2 (Total: 159, Up: 74 & Down: 85). The fasta file obtained as output for the custom transcriptome assembly is provided in Supplementary Information S3.

Volcano plots from DESeq2 and edgeR of differentially expressed genes in CHIKV-infected *Aedes albopictus* head/thorax compared with uninfected controls . DESeq2 was performed by aligning reads to the reference *Aedes albopictus* genome; while edgeR analysis was done on reads that did not align to the reference genome and were aligned to the custom transcriptome.

### 2.2. Ontology Analysis

**Figure 2:**
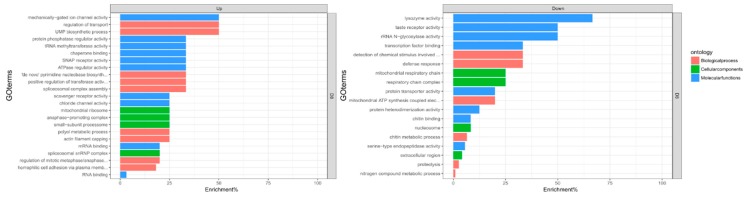
topGO enrichment comparison in differentially expressed genes. Enrichment analysis of up- and down-regulated genes in the heads and thorax of *Aedes albopictus* mosquitoes in response to CHIKV at 8 dpi. Enrichment percentage is calculated as the ratio of “significant” (number of times the gene ontology number was observed as differentially expressed) to “expected” (number of times the gene ontology number was expected based on observation in control samples) gene numbers.

Gene set enrichment analysis and ontology performed using topGO revealed that several biological and molecular processes were significantly modified during CHIKV infection (Figure 2). The complete output of the topGO analysis is provided in Supplementary Information S4.

In the head/thorax of *Aedes albopictus*, up-regulated molecular functions were RNA (*p*-value: 0.046) and mRNA binding (*p*-value: 0.049), and molecular functions that were down-regulated were lysozyme activity (*p*-value: 0.0038), serine-type endopeptidase activity (*p*-value: 0.0058), protein heterodimerisation (*p*-value: 0.00181) and chitin binding (*p*-value: 0.00532). Biological processes that were down-regulated were defence response (*p*-value: 9.70 × 10^−5^), proteolysis (*p*-value: 0.0075) and chitin metabolic process (*p*-value: 0.0106). Up-regulated biological processes were homophilic cell adhesion via the plasma membrane (*p*-value: 0.0056), UMP (uridine monophosphate) biosynthetic process (*p*-value: 0.0245), regulation of transport (*p*-value: 0.0245) and spliceosomal complex assembly (*p*-value: 0.0342). 

### 2.3. RT-qPCR (Reverse Transcriptase-Quantitative Polymerase Chain Reaction) Based Validation of RNASeq Data

Fourteen differentially expressed genes were selected, based on previously described involvement in the immune response, for validation using RT-qPCR (11 aligned to the reference genome and 3 from custom transcriptome) (Table 3). For this, heads/thoraxes were dissected from five individual CHIKV-infected adult *Aedes albopictus* female mosquitoes (8 dpi). Heads/thoraxes from uninfected mosquitoes were used as controls.

The RT-qPCR results, using gene-specific primers, compared with the RNAseq data (Table 3) showed that 7 out of 14 target genes were concordant.

### 2.4. Functional Significance of BTKi in CHIKV-Infected RML12 Cells

**Figure 3:**
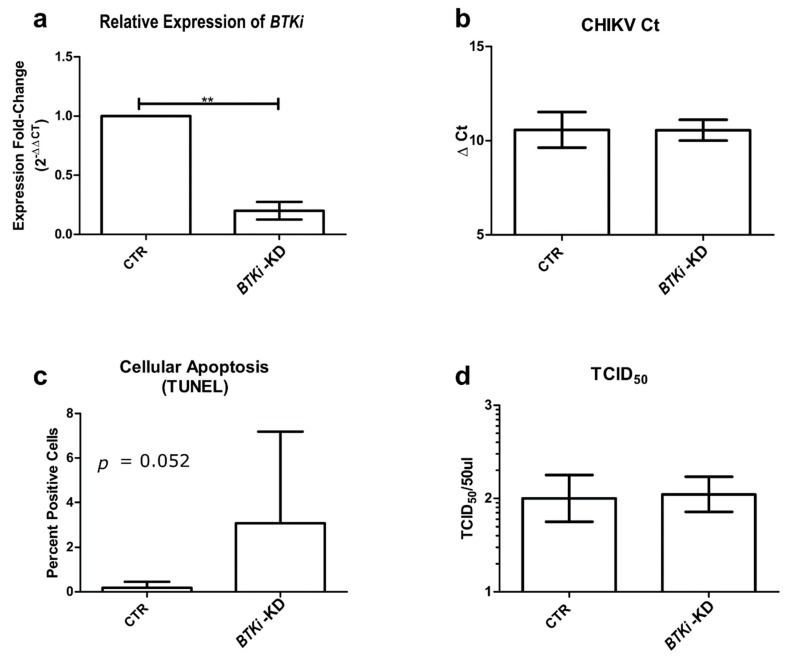
(**a**) Comparison of BTKi expression between BTKi-KD and GFP (green fluorescent protein)-KD RML12 cells; (**b**) Comparison of intracellular CHIKV RNA via RT-qPCR in BTKi and GFP knockdown RML12 cells; (**c**) Comparison of % of apoptosis detected in BTKi-KD and GFP-KD RML12 cells; (**d**) Comparison of TCID_50_ of extracellular CHIKV between BTKi and GFP knockdown RML12 cells.

Inhibitor of Bruton’s tyrosine kinase (*BTKi*) was identified to be one of the significantly up-regulated transcripts, both in RT-qPCR and RNASeq. To assess the functional significance of *BTKi*, an *Aedes albopictus* larval cell line (RML12) was transfected with anti-BTKi dsRNA. Twenty-four hours post transfection, the cells were infected with CHIKV (MOI: 1). Twenty-four hours post infection, the successful knockdown of *BTKi* gene was confirmed with RT-qPCR returning a ΔΔCt value of 2.39 (expression fold change: 0.1913, decrease in expression of *BTKi*: 80.87%). There were no significant changes in the viral RNA (ΔΔCt: −0.022, expression fold change: 1.015), or TCID_50_ (Figure 3b,d). The results indicate that inhibition of *BTKi*, while detrimental to host cells, may not have a direct impact on viral replication.

**Figure 4:**
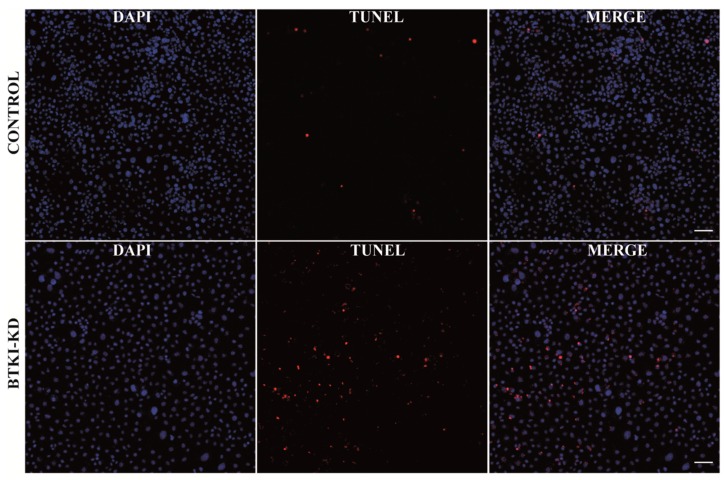
TUNEL staining of *BTKi* knocked down RML-12 cells 24 hours post infection with CHIKV showing increased apoptosis compared to control RML12 cells (Scale bar: 50 µM).

BTK has previously been shown to be involved in inducing apoptosis. To determine whether similar pathways are involved in CHIKV-infected mosquito cells, anti-*BTKi* dsRNA-transfected and CHIKV-infected RML12 cells were grown on glass cover slips and 24 hpi TUNEL (terminal deoxynucleotidyl transferase dUTP nick end labeling) staining was performed to quantify apoptosis. RML12 cells transfected with anti-GFP dsRNA and infected with CHIKV were used as controls. Cell nuclei were stained using DAPI and apoptosis was measured as the number of cells showing terminal deoxynucleotidyl transferase dUTP nick end labelling. The percentage of apoptosis was measured as the ratio of apoptotic cells to DAPI (4′,6-diamidino-2-phenylindole) -stained cells (i.e., nuclei) (Figure 4). Significant apoptosis was detected in BTKi-KD cells compared to control cells, 3.07% in BTKI-KD RML12 cells versus 0.186% in control RML12 cells (*t*: 2.0965, df: 16 and *p*: 0.0261) (Figure 3c).

## 3. Discussion

Multiple previous studies have been performed to understand the interactions between chikungunya virus and *Aedes aegypti*, the traditional vector [17,18,19]. Previously, RNASeq was performed on *Aedes albopictus* midguts 2 days post CHIKV infection, which identified a number of differentially expressed genes [16]. These results helped toward understanding the host–pathogen interaction at the first critical barrier site, the midgut. Yet, our understanding of transcriptome level interactions in *Aedes albopictus* after viral dissemination is preliminary. We attempted to study the virus–vector interaction in the head/thorax region 8 dpi as a proxy for the dissemination site. This site is important as successful infection of salivary glands (included in the thorax) ensures the presence of the virus in the saliva of the mosquito and in turn enables successful transmission.

Our results show differential regulation of biological processes including RNA and mRNA binding, lysosomal and serpin pathways and down-regulation of defensin genes. These could be due to either mosquito immune responses to CHIKV or viral modulation of immunity. Processes, such as the regulation of transport and homophilic cell adhesion via the plasma membrane, could be involved in viral assembly and export [20,21,22,23]. Functional studies to determine whether these genes are pro- or anti-viral are needed and could explain the role of these genes in the infection process. 

Interestingly, two odorant binding proteins (OBPs) were found to be differentially expressed. While Obp25 (AALF018602) was up-regulated, D7 protein (AALF024478) was down-regulated. In *Aedes aegypti*, salivary glands infected with dengue virus showed differential expression of odorant binding proteins [24]. It was shown that this down-regulation of OBPs reduced the chemosensory abilities of the mosquitoes, which may affect host-seeking and hence there is a reduced exposure to the virus via feeding, which may hinder transmission capabilities. Our results suggest that similar mechanisms may be in play here as well, although this remains to be verified with functional studies.

The RNAi pathway is one of the major pathways involved in antiviral responses in insects [25,26]. Consistent with our previous study on *Aedes albopictus* midguts post CHIKV infection, our data does not show statistically significant changes in the expression of genes involved in RNAi pathways in the head and thorax. This is consistent with a previous study involving the chikungunya virus and *Aedes aegypti* [17]. While it is possible that regulation of the RNAi pathway does not occur at the transcriptional level or that RNAi proteins are not differentially regulated but ubiquitously expressed, more focused validation may provide a clearer conclusion to this observation. Expression levels at different time points post infection may also need to be assessed before reaching this conclusion. The results are also consistent with other known antiviral genes like *JAK/STAT, IMD* and *Toll* pathways, which were also not found to be differentially regulated [17].

Our results showed that the concordance between our RNASeq and RT-qPCR data was low (50%). This was possibly because there were not enough biological replicates for RNA-Seq analysis. Higher concordance can be achieved by increasing the number of infected and control samples. It is possible that due to the low number of replicates and reduced statistical power, some differentially expressed transcripts could be missed. Also, to be noted is the fact that tissue from six mosquitos were pooled for testing. There could be biological variation, both in terms of infection status and transcriptional response of the mosquitos. Hence, the RT-qPCR-based validation was performed on individual mosquitoes. This variation in pools could also explain the reduced concordance between RNASeq and RT-qPCR. For functional analysis, further research was focused on *BTKi*, which was validated through RT-qPCR. 

BTKi is an inhibitor of Bruton’s tyrosine kinase (Btk) [27]. Btk is heavily involved in innate immune responses [28]. In higher level organisms, inactivating mutations in Btk results in a condition known as X-linked agammaglobulinemia (XLA), characterised by the inability to produce mature B-cells and gamma globulins (including antibodies). Increased susceptibility to apoptotic death on exposure to pathogen-associated inflammatory signals is observed in BTK-deficient macrophages. BTK is involved in NK cell activation via the up-regulated NF-κB (nuclear factor kappa-light-chain-enhancer of activated B cells) pathway [29]. Btk, along with Tec kinases, are heavily involved in osteoclast differentiation and regulation. Severe osteopetrosis is observed in mice with a non-functional *Btk* gene. Reduced osteoclastic bone resorption was observed in osteoporosis and inflammation-induced bone destruction on inhibition of Tec kinases. Btk and especially Tec kinases, are selectively expressed in osteoclasts and not in osteoblasts. Increased bone mass in mouse without Btk is due to defective osteoclastic bone resorption because of defective osteoclast differentiation [30]. CHIKV is known to infect osteoblasts and up-regulate IL-6 (interleukin-6) and RANKL (Receptor activator of nuclear factor kappa-Β ligand) generation and decrease OPG (osteoprotegerin) production. An altered RANKL/OPG ratio gives rise to arthralgia, a characteristic morbidity of chikungunya fever [31]. Literature also presents evidence of the benefit of Btk inhibition in viral inflammatory disease. Specifically, BTK inhibition with ibrutinib has shown a major protective effect in the lung tissue of mice during influenza viral infection by ameliorating excessive inflammatory response [32]. 

Our data suggests that BTKi is significantly upregulated in the head and thorax tissue of *Aedes albopictus* mosquitoes after chikungunya virus infection. While the knock-down of *BTKi* in RML12 cells did not result in significant differences in viral titers or RNA levels, there was increased apoptosis in cells. These observations are consistent with a recently published study that showed that inhibition of BTK is protective in mammalian host tissue during influenza viral infection [32]. In the context of CHIKV-induced arthritis in patients, it is interesting to note that BTK is involved in osteoclastogenesis and the inhibition of BTK on bone resorption is protective. Our data suggests a possible conserved mechanism at play for BTK and associated Tec kinases in the context of chikungunya infections in both mosquitoes and mammals.

Overall, our results showed significant changes in the transcriptome of *Aedes albopictus* mosquitoes after CHIKV dissemination. This study, for the first time, examined differential gene expression in the head and thorax (a dissemination site containing salivary glands) of infected mosquitoes. Our results can be utilized for determining potential proviral and antiviral host factors, and in turn, will be helpful in reducing the high impact of CHIKV infections by targeting the vector, *Aedes albopictus*.

## 4. Materials and Methods 

### 4.1. Chikungunya Virus and Aedes Mosquito Rearing, Infection, RNA Extraction and cDNA Preparation

*Aedes albopictus* mosquitoes were grown in the BSL-3 (Bio-Safety level-3) insectary at the Australian Animal Health Labs under the following conditions: 27.5 °C and 70% in relative humidity with a 12-h light and dark cycle from eggs obtained from Hammond Island in the Torres Straits. The mosquitoes used for experiments were from multiple generations (between 5–15 generations after collection). Chikungunya virus isolated in 2006 from a viremic patient, who returned from a trip to Mauritius, at the Victorian infectious disease reference laboratory, Melbourne, was used for this study. Our previous studies showed that this was a viral isolate from an ECSA clade (IOL sub-lineage), containing the E1-226 variation with improved fitness to *albopictus* mosquitoes and was closest in sequence similarity to the LR2006 isolate [16]. Five-to eight-day-old female mosquitoes were infected with CHIKV (1:100 dilution of stock; final TCID_50_: 1.5 × 10^7^/mL) spiked in fresh (not more than 2 days post bleeding) whole heparinized chicken blood collected from specific pathogen-free chicken collected on-site, through the Hemotek membrane feeding systems for one hour. Post feeding, blood-fed mosquitoes were separated and maintained in 200 mL cardboard containers at 27.5 °C, 70% humidity and in a 12:12 day:night photoperiod for 8 days with a 10% sugar solution ad libitum. For controls, chicken blood mixed with media supernatant from an uninfected Vero cell culture was fed to female mosquitoes. Eight days post exposure to CHIKV, the heads and anterior one-third of the thorax of the mosquitoes were dissected using 18-gauge needles, and tissues from six mosquitoes each were pooled into tubes, each with 50 µL of Qiagen RLTplus buffer (Qiagen, Chadstone, VIC, Australia) along with 5–10 silica beads (1 mm) and stored at −80 °C.

Tissue homogenisation was performed on a MP Biomedicals FastPrep −24™ homogeniser (MP Biomedicals Australia Pty Ltd, Seven Hills, NSW, Australia) (3 cycles, speed: 6.5 m/s, 45 s/cycle) via bead beating. RNA was then extracted using the RNeasy™ kit (Qiagen, Chadstone, VIC, Australia). Complementary DNA was generated by using random hexamers and Superscript-III reverse transcriptase (Thermo Fisher Scientific Inc., Scoresby, VIC, Australia) following the manufacturer’s protocols. cDNA generated from the RNA was tested for CHIKV RNA using an in-house-designed qRT-PCR, using E1 gene specific primers (Supplementary Table S1). For RNASeq data validation, adult *Aedes albopictus* female mosquitoes were infected with CHIKV as described above. RNA was extracted from the HTs of five infected mosquitoes 8 dpi, and cDNA was generated individually using previously described protocols. cDNA from the head/thorax of five uninfected mosquitoes was used as controls. 

### 4.2. RNASeq and RT-qPCR

RNASeq libraries were prepared using Nugen’s Ovation Universal RNASeq kit (Tecan Genomics, Inc, Redwood City, CA, USA) following the manufacturer’s specification with a minor modification in the HL-dsDNAse treatment. HL-dsDNase from Thermo Fisher Scientific was used in our library preparation, along with the 10× buffer supplied that their protocol used. The libraries were pooled and sequenced on a single lane of Hiseq-2500 (Macrogen Inc., Seoul, South Korea) to generate 2 × 100 bp reads. The fastq files were deposited in NCBI’s (National Center for Biotechnology Information) Sequence Read Archive (SRA Accession ID: SRP140387).

Gene-specific primers were designed for the 14 targets to be validated, and using 18s rRNA as an internal control, qPCR-based validation was performed on an Applied Biosystems QuantStudio™ 6 using the SYBR Green Master Mix: SYBR Premix Ex Taq II (Tli RNase H Plus) (Takara- Scientifix Pty Ltd., Clayton, VIC, Australia) (cycling conditions: 30 s at 95 °C, 40 cycles of 5 s at 95 °C and 30 s at 60 °C and a melt curve). Threshold cycle values were automatically calculated on the QuantStudio™ Software (Thermo Fisher Scientific Inc., Scoresby, VIC, Australia). ∆∆Ct values were calculated using the average ∆Ct value of controls and 18s rRNA as a reference. All primer sequences are provided in Supplementary Table S1.

### 4.3. Differential Gene Expression and Gene Ontology Analysis

Trimmomatic v0.36 was used for quality trimming of raw sequences [33]. Using Hisat2 v2.0.5, the reads were aligned to the CHIKV reference sequence (GenBank ID: MH229986) to assess the infection status [34]. The reads were aligned to the *Gallus gallus* genome and the aligned reads were removed bioinformatically before proceeding to the next step. This was performed to remove any RNA reads that could be from undigested chicken blood meal given to the mosquitos during infection. The reads were then aligned to the *Aedes albopictus* Foshan strain genome sequence (AaloF1) from Vectorbase (www.vectorbase.org) using Hisat2 [35]. The sorted Bam files were uploaded into Galaxy virtual lab v1.4.6.p5 and aligned transcripts were quantified using featureCounts [36]. Using default parameters, DESeq2 v2.11.38 was used to obtain differentially expressed genes [37].

A custom de novo transcriptome was generated using Trinity v2.3.2, by combining the unaligned reads [38]. edgeR was used to align the reads, measure the transcript counts and quantify the differentially expressed genes using the custom transcriptome [39]. BlastX and BlastN were used to annotate the genes [40,41].

Using topGO and based on *p*-values (Fisher’s exact method) differentially expressed genes were grouped based on their ontologies [42]. Enrichment percentage was calculated as the ratio of the number of times genes in the pathway were differentially expressed compared to the expected number by chance.

### 4.4. BTKi Knockdown and Functional Studies

Anti-BTKi dsRNA was generated by using a 422-bp-long segment (including the T7 promoter tag) of the coding sequence as a template via the Invitrogen™ MEGAscript™ RNAi Kit (Thermo Fisher Scientific Inc., Scoresby, VIC, Australia) by following the manufacturer’s protocol (reaction incubation time: 4 hours) (primer details in Supplementary Table S1). RML12 cells were grown in a modified Leibovitz’s L-15 medium (for 400 mL of L15 medium, 65 mL of FCS (fetal calf serum), 40 mL of TPB (Tryptose Phosphate Broth), 1.25 mL of Pen-strep and 5 mL of glutamine were added). Cells were maintained at 27 °C with 1% CO2.

RML12 cells of albopictus origin [43,44,45], grown in 24-well plates, were transfected with anti-BTKI dsRNA using Cellfectin® II (Thermo Fisher Scientific Inc., Scoresby, VIC, Australia) by following the manufacturer’s protocol. Briefly, cells were incubated for 5 hours with a mixture of 800 ng of purified dsRNA, 8 µL of Cellfectin® II reagent and 240 µL of transfection media (L15 medium with TPB and glutamine but no FCS and antibiotics) per well. The mixture was replaced with normal L15 growth medium at the end of incubation. For controls, RML12 cells were transfected with anti-GFP dsRNA.

Twenty-four hours post transfection with dsRNA, the cells were infected with CHIKV (MOI: 1, stock TCID_50_: 1.5 × 10^7^/mL). Briefly, each well (in a 24-well plate) of RML12 cells were incubated for one hour with 250 µL of infection medium (L15 medium with TPB and glutamine but no antibiotics and 2% FCS) and CHIKV. At the end of incubation, the infection media was replaced with regular L15 growth media. 

Twenty-four hours post infection, the cell culture supernatant was collected and TCID_50_ was performed on Vero cells. RNA was extracted by lysing the monolayer of RML12 cells with 350 µL of RLT Plus buffer. RNA extraction and cDNA generation were performed as described before. qPCR was performed to assess the knockdown of BTKi and change in viral RNA.

### 4.5. Confocal Microscopy and TUNEL Staining

For detection of apoptosis, RML12 cells were grown on glass cover slips (13 mm diameter) compatible with 24-well plates. The cells were transfected with anti-BTKI dsRNA or anti-GFP dsRNA (for controls) followed by CHIKV infection, as described before. BD Cytofix/Cytoperm™ (BD Biosciences, North Ryde, NSW, Australia) was used to fix the cells for one hour and stored in PBS at 4 °C.

TUNEL (terminal deoxynucleotidyl transferase dUTP nick end labelling) staining was performed using an *in situ* cell death detection kit, TMR red (Sigma-Aldrich, Castle Hill, NSW, Australia) and the nuclei was stained with 4′,6-Diamidine-2′-phenylindole dihydrochloride (DAPI) (Sigma-Aldrich, Castle Hill, NSW, Australia). The cells were then imaged in a Zeiss LSM 800 confocal system (Carl Zeiss Pty Ltd, North Ryde, NSW, Australia) using 10× objective covering at least 500 cells per image. Percentage of TUNEL positive apoptotic cells against the total number of cells (DAPI stained nuclei) was quantified using ImageJ (particle analyser plugin, https://imagej.nih.gov/ij/index.html) from each image. 

## Figures and Tables

**Table 1:** RNASeq data summary.

	Total Reads (millions)	% Mapped to Refseq Genome	% of CHIKV Reads
Infected HT1	47.15	63.28%	1.12
Infected HT2	40.60	60.35%	2.09
Control HT	42.10	62.91%	0.00

Total reads obtained from HT (Head and Thorax), their alignment percentage to the reference *Aedes albopictus* genome and the percentage of reads aligned to the chikungunya virus genome.

**Table 2:** Differential Gene Expression analysis.

	No of Differentially Expressed Genes		
	Total	Up	Down
DESeq2	96	51	45
edgeR	63	23	40

Number of genes found to be differentially expressed in mosquito head/thorax 8 dpi using DeSeq2 (*p*-value: <0.05) and edgeR (FDR: <0.05) analysis.

**Table 3:** List of genes validated using qRT-PCR and a comparison of the expression fold change between RT-qPCR and RNASeq.

	Gene Annotation	Expression Fold Change (RT-qPCR)	LogFC (RNASeq)
1	Quaking protein A	0.8(↓)	2.54
2	PDZ and LIM domain protein 7-like isoform X1	0.43(↓)	1.53
3	Putative ecdysteroid-regulated 16 kDa protein	0.96(↓)	2.07
4	Fat-like cadherin-related tumor suppressor homolog	0.01(↓)	3.51
5	Peptidylprolyl isomerase	0.75(↓)	2.24
6	Fasciclin-2-like isoform X1	2.97(↑)	3.55
7	Putative glycine-rich RNA binding protein	0	2.96
8	Leucine-rich immune protein (Long)	3.65(↑)	−1.65
9	Inhibitor of Bruton tyrosine kinase	17.54(↑)	2.49
10	Phosrestin i (arrestin b) (arrestin 2)	1149.59(↑)	1.51
11	Putative uncharactarised protein containing CysCysHisCys (CCHC) zinc finger domain	0.09(↓)	−3.12
12	Protein no-on-transient A isoform X2	0.01(↓)	−5.08
13	Ficolin-3-like	0.07(↓)	−6.42
14	PDZ and LIM domain protein Zasp-like isoform X5	2.9(↑)	10

List of genes selected for validation by qRT-PCR and their annotation based on BlastX and BlastN and results of RT-qPCR on the 14 gene targets selected from RNAseq DGE analysis. Expression fold change for RT-qPCR was calculated using 2^−ΔΔCt^ and the up- or down-regulation compared to controls is indicated by arrows, while LogFC (from RNA-Seq data) was calculated using DESeq2 and edgeR.

## Supplementary Materials

The following are available online at https://www.mdpi.com/2076-0817/8/3/132/s1, S1: List of primers, S2: List of differentially expressed genes, S3: Custom transcriptome, S4 topGO output.
