# Supplementary material for: Whole Transcriptome Analysis of Aedes albopictus Mosquito Head and Thorax Post-Chikungunya Virus Infection
Source: Pathogens. 2019 Aug 27;8(3):132. doi: 10.3390/pathogens8030132 (PMC6789441; doi:10.3390/pathogens8030132)
Supplement: Supplementary file 1 [file pathogens-08-00132-s001.zip › supp data/S1 List of primers.docx]

Supplementary information for:

# Whole transcriptome analysis of *Aedes albopictus* mosquito head & thorax post-chikungunya virus infection

# Table A: List of Primers

| Primer | Sequence |
| --- | --- |
| CHIKV_E1_FOR | AAGAGCGATGAACTGCGCCGTAG |
| CHIKV_E1_REV | CTGGTACCTCGCATGACATGTC |
| AALF004300_D8_FOR | CACGAACCGAACGGATATG |
| AALF004300_D8_REV | TTCTGCTGCTGCTGTTG |
| AALF008354_D8_FOR | CGTGTCCTTCTACCAAATCC |
| AALF008354_D8_REV | TCTTGCGCTTCAGCTTAC |
| AALF011899_D8_FOR | GCTCGATGCGAGATAAGAAG |
| AALF011899_D8_REV | CTCGGTATCTTCAACGGTAATC |
| AALF012324_D8_FOR | GGCACCATCAATAGGGTAAC |
| AALF012324_D8_REV | CCTTCGAGCCAAACTCTATG |
| AALF012634_D8_FOR | CGGGAGATTTACGAGGTTTC |
| AALF012634_D8_REV | GCGTTCTTCCCTTCATCTC |
| AALF016505_D8_FOR | GGAATGTGGCAATGTGAATAC |
| AALF016505_D8_REV | GATCACTCGATCGGCATAAG |
| AALF016704_D8_FOR | ACTGACGTTCCCTTCAAAC |
| AALF016704_D8_REV | GCTCGATCGACTTCATCTTC |
| AALF021910_D8_FOR | GTGAACCTCATTCCGGATAC |
| AALF021910_D8_REV | CTTTCACCTCGGTCCAATC |
| AALF023547_D8_FOR | GAACCATCGTGGAAGAAGAG |
| AALF023547_D8_REV | CATCAGCTTCAGAGCCATATC |
| AALF025245_D8_FOR | CGGAGAAGCAGTTGGTATTC |
| AALF025245_D8_REV | CGATCGAGTGGAAGTCTTTG |
| AALF026574_D8_FOR | GAGGTGCTCGTTACATCTTG |
| AALF026574_D8_REV | GCAATGGGTGGTACCTTATC |
| DN129476_D8_FOR | GTGCTCTTCCGATCTTTCTC |
| DN129476_D8_REV | GAAGGTATCGTCGAGTTCAAG |
| DN131737_D8_FOR | GGGTCTTGAGCGAATGTATC |
| DN131737_D8_REV | TGCCGCATACTTGTAGTTATC |
| DN131885_D8_FOR | ACTCTGGTGTACCCTTATCC |
| DN131885_D8_REV | TTCCTAGTGGTAGTGATCGAG |
| 18srRNA_Fwd | CGGCTACCACATCCAAGGAA |
| 18srRNA_Rev | GCTGGAATTACCGCGGCT |
| BTKi_dsRNA_FWD | *GTCATAATACGACTCACTATA GGGAGA*TGTTCACGATTTGAGCTTCG |
| BTKi_dsRNA_REV | *GTCATAATACGACTCACTATA GGGAGA*AGATCTGGGTAGGCATCACG |
